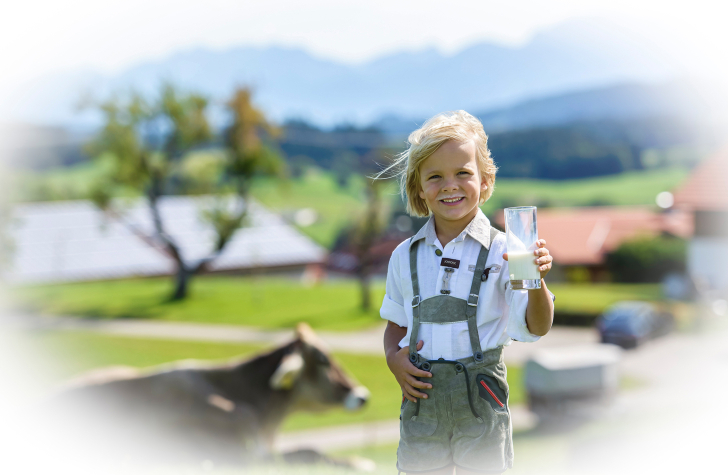# Diagnosis and Rationale for Action against Cow's Milk Allergy (DRACMA) Guidelines update - III - Cow's milk allergens and mechanisms triggering immune activation

**DOI:** 10.1016/j.waojou.2022.100668

**Published:** 2022-09-15

**Authors:** Sebastian A. Jensen, Alessandro Fiocchi, Ton Baars, Galateja Jordakieva, Anna Nowak-Wegrzyn, Isabella Pali-Schöll, Stefano Passanisi, Christina L. Pranger, Franziska Roth-Walter, Kristiina Takkinen, Amal H. Assa'ad, Carina Venter, Erika Jensen-Jarolim, On behalf of the WAO DRACMA guideline group

**Affiliations:** a Institute of Pathophysiology and Allergy Research, Centre of Pathophysiology, Infectiology and Immunology, Medical University of Vienna, Vienna, Austria; b University Clinics for Ear Nose and Throat, Medical University Vienna, Austria; c The Interuniversity Messerli Research Institute of the University of Veterinary Medicine Vienna, Medical University Vienna and University Vienna, Vienna, Austria; d Allergy Unit – Area of Translational Research in Pediatric Specialities, Bambino Gesù Children's Hospital, Rome, Italy; e Division of Pharmacology, Department of Pharmaceutical Sciences, Utrecht University, Utrecht, the Netherlands; f Department of Physical Medicine, Rehabilitation and Occupational Medicine, Medical University of Vienna, Austria; g Department of Pediatrics, NYU Grossman School of Medicine, Hassenfeld Childrens' Hospital, New York, NY, USA; h Department of Pediatrics, Gastroenterology and Nutrition, Collegium Medicum, University of Warmia and Mazury, Olsztyn, Poland; i Department of Human Pathology of Adult and Developmental Age, University of Messina, Italy; j VTT Technical Research Centre of Finland Ltd, Espoo, Finland; k Cincinnati Children's Hospital Medical Center, Cincinnati, OH, USA; l Childrenás Hospital Colorado, University of Colorado, Denver, CO, USA; m AllergyCare - Allergy Diagnosis Center Vienna, Private Clinics Döbling, Vienna, Austria

**Keywords:** Allergy, Cow's milk, Beta-lactoglobulin, Food allergy, Pasteurization

## Abstract

**Background:**

The immunopathogenesis of cow's milk protein allergy (CMPA) is based on different mechanisms related to immune recognition of protein epitopes, which are affected by industrial processing.

**Purpose:**

The purpose of this WAO DRACMA paper is to: (i) give a comprehensive overview of milk protein allergens, (ii) to review their immunogenicity and allergenicity in the context of industrial processing, and (iii) to review the milk-related immune mechanisms triggering IgE-mediated immediate type hypersensitivity reactions, mixed reactions and non-IgE mediated hypersensitivities.

**Results:**

The main cow’s milk allergens – α-lactalbumin, β-lactoglobulin, serum albumin, caseins, bovine serum albumins, and others – may determine allergic reactions through a range of mechanisms. All marketed milk and milk products have undergone industrial processing that involves heating, filtration, and defatting. Milk processing results in structural changes of immunomodulatory proteins, leads to a loss of lipophilic compounds in the matrix, and hence to a higher allergenicity of industrially processed milk products. Thereby, the tolerogenic capacity of raw farm milk, associated with the whey proteins α-lactalbumin and β-lactoglobulin and their lipophilic ligands, is lost.

**Conclusion:**

The spectrum of immunopathogenic mechanisms underlying cow's milk allergy (CMA) is wide. Unprocessed, fresh cow's milk, like human breast milk, contains various tolerogenic factors that are impaired by industrial processing. Further studies focusing on the immunological consequences of milk processing are warranted to understand on a molecular basis to what extent processing procedures make single milk compounds into allergens.

## Introduction

Cow's milk has many different faces. This becomes most apparent for the consumer in the dairy products section of a supermarket, or when a young mother is in need of alternatives to breastfeeding her baby. Dairy products as a major staple food worldwide must not only meet the hygienic standards, be storable, transportable, and accessible to people globally, but also be adapted to settings where cold chains are difficult to manage or entirely unfeasible, due to geography and climate conditions. This requires multiple processing and conservation steps and consequently the milk industry today is a highly specialized discipline. We increasingly understand that industrial processing, especially the acidification and heating of the milk through pasteurization and sterilization, affects the composition of cow's milk in terms of fat, carbohydrates (such as in lactose free milk), vitamins, and proteins, the latter especially in terms of their tertiary protein structure (Fig. 1), and their quaternary state due to aggregation of homo- and heteromeric complexes, which is only to some degree counteracted by homogenization. The complexity of products can be seen alongside the complexity of possible hypersensitivity reactions. Here, we will mostly focus on **immediate type reactions to milk caused by IgE antibodies**, relevant in oral allergy syndrome, acute and contact urticaria, angioedema, gastrointestinal symptoms, and anaphylaxis.^1^ But, we will also address **mixed hypersensitivity reactions** involving besides IgE also T-cells and eosinophils that foster delayed inflammatory symptoms such as exacerbation of atopic dermatitis, eosinophilic esophagitis and eosinophilic enterocolitis, rather tending to chronicity of the symptoms (Table 1).^2^ In addition, there are **non-IgE mediated types of hypersensitivities** to milk, which are in terms of the causative immune mechanisms partly, but yet not completely understood, like food protein-induced allergic proctocolitis (FPIAP), the food protein-induced enterocolitis syndrome (FPIES), food protein-induced enteropathy (FPIE), and Heiner Syndrome. T-cell mediated contact dermatitis to milk is rare. Interestingly, cow's milk allergy (CMA) is associated with dysbiosis and increased susceptibility for infections, and it has been suggested that it can be managed (in part) by pro-, pre- and symbiotics.^3^^,^^4^ In contrast, consumption of raw farm milk, just as breast milk, has been associated with protective effects against allergies, asthma, atopic eczema and infections.^5^ As raw milk may contain hazardous pathogens like mycobacteria, Salmonella or Brucella, heat inactivation – pasteurization – has been developed. Below we will analyze the differences between raw milk and processed milk to understand the immunological mechanisms behind milk-related adverse reactions. In all milk allergy syndromes, however, the protein fraction of milk, ie, cow's milk proteins (CMP), are the causative agent, while additive factors such as vitamins, plant flavonoids, iron complexes, omega-3 fatty acids, or butyrate, may play an anti-inflammatory, protective role.

**Table 1:** Classification of food allergic reactions. According to Sampson et al. 2016^118^ under CC Y 4.0 license (http://creativecommons.org/licenses/by-nc-nd/4.0/), slightly modified.

IgE-mediated	Mixed IgE and Non-IgE-mediated	Non-IgE-mediated
**Skin**		
Urticaria and contact urticaria	Atopic dermatitis	Dermatitis herpetiformis Duhring (DHD)
Angiodema		Contact dermatitis
Erythematous-morbilliform rash		
Flushing		
**Respiratory**		
Allergic rhinoconjunctivitis	Asthma	Food-induced pulmonary hemosiderosis (Heiner's Syndrome)
Acute bronchospasm		
**Gastrointestinal**		
Oral Allergy Syndrome	Eosinophilic esophagitis (EOE)	Food protein-induced entero-colitis syndrome (FPIES)
Acute gastrointestinal spasm	Eosinophilic gastritis	Food protein-induced proctocolitis syndrome (FPIES)
	Eosinophilic gastroenteritis	Celiac disease
**Cardiovascular**		
Dizziness and fainting		
Anaphylaxis		
Food-associated, exercise-induced anaphylaxis		
**Miscellaneous**		
Uterine cramping and contractions		
Feeling of pending “doom”		

## The encounter of bovine milk: antigen or tolerogen

### Cow's milk proteins and protein fragments in formula

The child's first contact with milk is typically related to breastfeeding, which provides the essential nutrition source during the first months of life. It supports growth and development and also the child's defense against infections by providing antimicrobial and immunomodulatory factors from the mother, while the child's own immune system is mounting.^6^^,^^7^

In most of the food cultures around the world, infant formula based on cow's milk is the most suitable alternative to human milk whenever breastfeeding is not possible. Importantly, some differences exist between human and bovine milk regarding protein composition; for instance, human milk does not contain any equivalent to β-Lactoglobulin (BLG) (Table 2), unless the breastfeeding mother consumes cow's milk or other milks containing BLG. Notably, to produce infant formula, cow's milk is additionally heavily processed and often supplemented and fortified (see below).

**Table 2:** Compositions of human and cow's milk, modified after Crittenden et al.^119^ and Villa et al.^81^

Milk fraction	Protein family	Protein	Human (mg/ml)	Cow (mg/ml)	Allergen name^a^	Impact
Caseins	Caseins	**α-s1-casein**	0	11.6	**Bos d 9**	**major**
		**α-s2-casein**	0	3.0	Bos d 10	minor
		**β-casein**	2.2	9.6	**Bos d 11**	**major**
		**κ-casein**	0.4	3.6	Bos d 12	minor
		**γ-casein**	0	1.6	Bos d 8	
Whey proteins	Lipocalins	**β–lactoglobulin (BLG)**	0	3.0	**Bos d 5**	**major**
	Lysozymes	**α-lactalbumin (ALA)**	2.2	1.2	**Bos d 4**	**major**
	Transferrins	lactoferrin	6.0	0.1–0.2	–	(evtl.minor)
	Albumins	serum albumin	0.4	0.4	Bos d 6	minor
	Immunoglobulins	immunoglobulins	0.8	0.6	Bos d 7	minor
		other	0.8	0.6		

a According to the official WHO/IUIS nomenclature^49^ allergens are abbreviated by the first letters of the genus in Latin, followed by the first letters of the species and a number, which represents the chronological order of their discovery. Bos d: abbreviation of Bos domesticus

There are, however, great regional differences in the composition of infant formulas depending on the need and timeframe for when the formula will be required. Preterm babies are usually fed with bovine-derived milk fortifiers (BMF) which contain CMP.^8^ It has been shown that bovine colostrum fortifier (BCF) is a better alternative to the highly processed mature BMF.^9^

For instance, in China, bovine lactoferrin is added to formulas as additional supply of iron for mimicking the higher levels of lactoferrin in human milk and providing the same level of antibacterial activity. This raised the need for specific analytical methods to identify lactoferrin in hydrolyzed formulas.^10^ Babies fed with infant formula milk are often introduced to complementary food of any kind earlier than exclusively breastfed infants.^11^ This is of importance as immunosuppressive agents are missing in milk formulas, because they are destroyed during the hydrolyzation process. Therefore, research efforts have been made to supplement bovine transforming growth factor-β (TGF-β) to CMF, because TGF-β caused oral tolerance in a mouse model.^12^ Interestingly, human milk-derived fortifiers (HMF) have recently become available, but compared to BMF did not decrease the risk for necrotizing enterocolitis in breastfed preterm infants according to a Cochrane database research.^13^ The criteria for the composition of infant formula have been defined,^14^ and CMP content is expressed as amount total protein (g/100 kcal), independent of whether it contains intact or hydrolyzed milk proteins.

### Industrial processing makes a difference regarding the cow's milk proteins

The prevalence of cow's milk protein allergy (CMPA) has been stable over the past century, ranging from 2 to 3%.^15^, ^16^, ^17^ Around the world, CMPA is predominantly a problem in young children, as it most often resolves in adulthood.^18^ The prevalence of CMPA could be related to modern lifestyle, such as working mothers, older age of mothers at birth, need for daycare, which all are factors related to the lack of availability of breastfeeding, and forced more babies into supplementation with cow's milk.

In addition, over time wet-nursing became less popular and less practicable in industrialized countries. For premature babies, human donor milk banks are increasingly established, but these human milks for hygienic reasons do require donor milk pasteurization, traditionally batch pasteurization (62–63 °C; 30 min). Novel processing of human milk is being developed such as thermal conditions (72–75 °C, 20–60 s) and non-thermal processing (high pressure processing, microwave irradiation).^19^ A recent Cochrane database review^20^ showed that artificial bovine-based infant formula milk rendered higher rates of weight gain and linear growth than donor milk. However, as compared to the remarkable benefits of human donor milk, artificial formula milk bears the risk of developing necrotizing enterocolitis.

In parallel, dairy farming has been significantly upscaled in terms of production per cow, and milk processing has been developed and refined to supply the growing population with safe nutrition. There are manifold different processes during milk manufacturing, customized to the desired end product.

Principally, whole milk is collected from farms, transported in a cooling chain, and stored as raw milk, until in the dairy plant the milk fat is separated from the aqueous phase by centrifugation. The farm milk may then be pre-pasteurized (called thermization, short heating above 60 °C) to achieve a longer storage potential before the final processing is initiated. The fat-free 0% milk is pasteurized to kill pathogenic bacteria. The milk fat is pasteurized as well at different temperatures before being homogenized into smaller particles and put back into the skimmed milk. By adding stored cream, any fat specification can be created, either no-fat milk, 2%, or 3.5% consumption milk.

The impact of heating depends on the combination of time and temperature. Pasteurization of milk is done at low temperature/long time, LTLT (62–63 °C, 30 min), but nowadays most cow's milk is pasteurized at short time/high temperature (STHT) (72–80 °C, 10–60 s). This type of milk is kept under refrigeration temperatures (<4 °C). Milk may then be clarified, and fortified with vitamins, mainly D3 (especially in Northern and English-speaking countries). Only then it is packed and delivered in a cooling chain to supermarkets (Fig. 1).

Processing can be widened through, for instance, filtration of the skim milk fraction to create milk with extended lifetime (ESL-milk). Besides milk pasteurization, milk is also sold as ultra-high heated milk (ultra-high temperature, UHT), where milk is heated for a very short time above 130–140 °C. This sterile packed type of milk can be stored at room temperature. Differences in heat processing are culture dependent. In the warmer regions of Southern Europe much milk is mainly being consumed as UHT milk, whereas in the middle and northern parts, milk is mostly sold as pasteurized milk.

For specific purposes milk can be fractionated and further processed. For instance, precipitation using hydrochloric acid (HCl) leads to a separation of the whey containing the soluble whey proteins which can be decanted, leaving the casein fraction. In the production of lactose-free milk, the milk is processed with the enzyme lactase to hydrolyze the disaccharide lactose into monosaccharides. All processed products can be dried and powdered in plants, starting with concentration steps using coagulators, centrifuges, heaters, followed by evaporators, blowers or cyclones in drying units, the milled powder is then packed.^21^ Therefore, multiple steps during milk processing significantly alter not only the composition of the constituents, but also the structure associated with their inherent biological function. Laboratory technologies, like nuclear magnetic resonance (NMR) determine how native milk proteins can become denatured in the different steps of processing, even though extrapolation to processes in the complex milk matrix may be difficult.^22^

Overall, the major ways of processing that severely modify the antigenicity of milk proteins due to denaturation and aggregation are: 1) acidification by HCl treatment, 2) defatting and homogenization associated with a loss of lipophilic ligands, and 3) heating in terms of pasteurization or sterilization process above 70 °C (drying, condensation or concentration processes) (Fig. 1).

### What makes a cow's milk protein allergenic?

During sensitization, cow's milk allergic patients form IgE to CMPs, targeting the casein protein family and/or the whey proteins β-lactoglobulin (BLG) and α-lactalbumin (ALA), or several minor proteins, such as lactoferrin, bovine serum albumin (BSA), or bovine immunoglobulins.^23^

Over the past decade it is better understood what makes a foreign protein offensive to the immune system, how children get sensitized, and which features of the processing contribute to the allergenic potential of the different milk proteins.

The signals relevant in allergy have recently been reviewed in a position paper of the European Academy of Allergy and Clinical Immunology (EAACI).^24^ Some allergens can directly stimulate the immune system by so-called allergen-associated danger signals; among them allergen-associated molecular patterns (AAMP) play a role in activating innate immune cells and initiating type 2 immune responses^25^ via a plethora of receptors and pattern-recognition receptors (PRR). In cow's milk CD14 occurs as a soluble pattern recognition receptor^26^ and is involved in the passive antibacterial defense sensing lipopolysaccharide (LPS) on Gram-negative bacteria, which may elicit mastitis in cows usually causing worse milk quality.^27^ Importantly, low CD14 levels in human mothers' milk were associated with atopic dermatitis in their babies at 9 months.^28^ It is not known whether milk processing affects the content of CD14.

CD14 facilitates the activation of Toll-like receptor-4 (TLR4) by LPS, which is relevant for some allergens. The house dust mite allergen Der p 2 is structurally homologous to part of the TLR4 signaling complex, the myeloid differentiation-2 molecule (MD-2)^29^^,^^30^ implicating interference with the innate defense arm. Also human milk may activate via its oligosaccharide 2′-fucosyllactose the TLR4-NFkB signaling pathway.^31^ It has not been investigated yet whether the oligosaccharide content in cow's milk, which depends on the cattle breed,^32^ relates to the allergenicity of milk. Recently, β-lactoglobulin was proposed as a novel player in the farm-protective effects against allergies.^33^ In this study, BLG was detected in cow stable dust and aerosolized up to 300 m around the farm; it was complexed with zinc and tightly associated with LPS, with well-known TLR4 signaling capacity. Other allergens with lipid-binding capacities similar to the lipocalin BLG, as apolipophorin, secretoglobins (Fel d 1^34^), 2S albumins, non-specific lipid-binding proteins (nsLTPs), and PR10 proteins may have the ability to amplify the LPS/TLR signaling pathway, too.^35^

Some allergens can stimulate protease-activated receptors (PAR)-2,^35^ which open tight junctions and subsequently lead to the disruption of the epithelial barrier function in allergic rhinitis.^36^ In human milk, IgA with its protease function has already been shown to activate PAR-2.^37^ Secretory immunoglobulins in cow's milk can act as IgE-binding minor allergens (Table 2).

Acute phase proteins (like ALA, BLG and Lf) are elevated in milk in mastitis, because they are involved in the innate immune defense of the cow.^38^, ^39^, ^40^

Some of the Th2 biasing properties of milk proteins have to do with their capacity to – by evolutionary homology, or by chance – interact with human immunomodulatory proteins or receptors. It seems clear that these characteristics critically depend of the allergen's tertiary molecular structures. In milk this seems to be especially important as it is significantly processed in industrial manufacturing, involving acidification, defatting and of course heating/pasteurization, as discussed above, and these processes alter the secondary, tertiary and quaternary structure (Fig. 1). While it is common sense that processing, especially hydrolysis, reduces IgE binding to conformational epitopes of milk proteins associated with a reduction of allergenicity, recent studies proposed that processing of milk may also correlate with a higher sensitization capacity or allergenicity.^41^, ^42^, ^43^

The glycosylation status renders proteins very stable against physical forces and resistant to gastrointestinal proteolysis, but in dairy cows, post-translational modifications of proteins differ depending on the cattle breeds and feed.^44^^,^^45^ The aggregation state of allergens, ie, their quaternary structure, determines their allergenicity and immunogenicity potential. It has been shown that the increasing aggregation state of BLG and of ALA by pasteurization, in contrast to cooking, increase the likelihood for uptake via Peyer's patches, checkpoints for active immune stimulation especially by particulate antigens, instead of non-immunogenic nutritional absorption by intestinal epithelial cells.^41^

## Structural, functional, and allergenic characteristics of single cow's milk proteins

Heating of milk causes multiple changes. During heating most of the bacteria in milk are destroyed, but also the concentration and functionality of proteins, especially of the heat sensitive whey proteins, are diminished. Furthermore, heating changes the concentration of peptides.

### Proteins of the whey fraction of milk

Whey is an important by-product of cheese processing. Whey proteins can be harvested after acidic coagulation of milk from the supernatant. They harbor bacteriostatic functions, which can be impaired by milk processing, especially pasteurization, because whey proteins are heat-sensitive. Milk samples heated for 30 min below <75 °C suppressed the growth of *Streptococcus thermophilus*, *Lactococcus lactis* and *Pseudomonas fluorescens*, whereas samples heated above 75 °C did not. Differences can in part be due to the denaturation of bovine gamma-globulins with antibacterial specificities.^46^ Therefore, the anti-infective properties of raw farm milk are lost due to denaturation of whey proteins after pasteurization, similarly to its anti-allergy properties.^5^ The effects on proteins are complex: Heating up to 72 °C has been demonstrated to cause aggregation of ALA and BLG, where above this temperature the proteins are destroyed.^41^ Similarly, heating above 72 °C affected the bioactivity particularly of lactoferrin and bovine immunoglobulins compared to BSA.^47^ Also the enzymatic activity of alkaline phosphatase (ALP) is lost upon pasteurization and therefore serves as an indicator for bacteriologically safe milk. The Tubercle bacteria are already killed below the temperatures used to inactivate ALP.

### Alpha-lactalbumin – ALA

In people and cattle, alpha-lactalbumin (ALA) has 123 amino acid residues with homology to the lysozyme family and a very similar 3D structure, with a large alpha-helical domain, connected with the Ca^2+^-binding loop to a smaller β-sheet domain (pdb 1F6S) (Fig. 2). Ca^2+^ stabilizes the fold. Bioactive peptides derived from ALA have multiple functions, like opioid agonism, ACE inhibition, stimulatory effect on the ileum.^48^ When ALA induces and binds IgE, hence acting as allergen, it is termed *Bos d 4* which represents a major milk allergen (Table 2).^49^

**Fig. 2:**
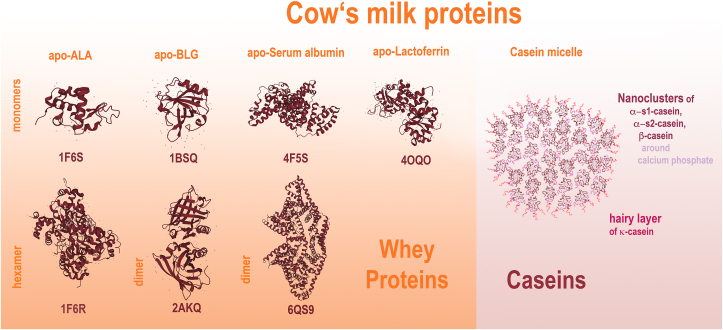
**Molecular milk allergens. Left panel, Whey proteins**: apo-α-lactalbumin, apo-β-lactoglobulin, apo-serum albumin and apo-lactoferrin may occur as monomers, or oligomers. Binding of ligands in the holo-variants of these proteins may change their oligomeric state and tolerogenic potency. Below each molecule the pdb accession number is given. Bovine immunoglobulins are not illustrated. **Right panel, Caseins:** Schematic model of a casein micelle according to Ref. ^22^, consisting of all caseins. Usually, α-s1-, α-s2-, and β-caseins cluster around amorphic calcium phosphate, thereby assembling nanoclusters, while κ-casein is found on the border zone. Processing may change the protein tertiary and quaternary structures as well as the composition of a micelle

ALA is a heat-stable bioactive whey protein; it is a natural regulator for lactose biosynthesis. ALA can interact with lipid membranes, proteins and low molecular substrates like peptides, which depends on its metal binding. ALA has a single strong Ca ^2+^ binding site, which also binds Mg^2+^, Mn^2+^, Na^+^, and K^+^, and in addition it has several Zn^2+^ binding sites. The supplementation of infant formulas with bovine ALA did not support uptake of iron.^50^

The structure of ALA can be affected also by addition of unsaturated C18 fatty acids such as oleic (18:1), linoleic (18:2), and α-linolenic (18:3) acid. ALA linked to oleic acid has been shown to acquire bactericidal and cytotoxic anti-tumoral activities,^51^ especially when thermally unfolded and stabilized by oleic acid.^52^ This apoptotic complex has been termed Human Alpha-Lactalbumin Made Lethal to Tumor cells (HAMLET), and in bovines accordingly BAMLET. However, more recent studies have shown that the cytotoxic effect of these complexes depends on oleic acid rather than ALA.^38^ As HAMLET is secreted into human breast milk, its occurrence in frozen human donor milk stored for critically ill and preterm babies should be critically investigated, given its potential capacity to trigger inflammation in the sensitive neonates.^52^

Linoleic acid has been shown to promote a Th2 environment^53^ and complex formation of ALA, but also BLG with linoleic acid leads to aggregation, enhanced IgE-binding and degranulation capacity.^54^ In contrast, processing modifications like glycation, phosphorylation and acetylation reduce the allergenicity of these complexes.^55^

An additional phenomenon occurring in the complex milk matrix during processing is that ALA thermally co-aggregates with the heat-sensitive lactoferrin, accompanied by an increase in β-sheet content and decrease in α-helix content.^56^ Dimers of ALA are found in raw milk (pdb 1F6R), but dry heating leads to extensive dimer and aggregate formation of ALA,^57^ in pdb a hexameric variant is depicted (1F6R) (Fig. 2). Therefore, heating not only changes the tertiary, but also the quaternary state of ALA. This is a problem in terms of a change of immunogenicity by heating and baking of milk.

When ALA is thermally precipitated during fractionation of whey proteins, its fold can almost be reconstituted by pH adjustment, while BLG irreversibly aggregates upon heat treatment, but not upon acidification.^58^ However, extensive cooking above >72 °C also destroys ALA.

### Beta-lactoglobulin – BLG

The main whey protein is BLG with a concentration of up to 60% of the total whey protein fraction.^59^^,^^60^ BLG is another bioactive compound, and with 162 amino acid residues it is similar in size to ALA. It folds into a sole α-helix and a β-barrel, with a central hydrophobic pocket (pdb 1BSQ) (Fig. 2). When its structure changes and IgE antibody binding occurs, BLG is termed *Bos d 5* and represents a major cow's milk allergen. BLG is mainly dimeric under conditions typically present in milk (pH 3–7) (pdb 2AKQ) (Fig. 2), and the dimers occur in a dynamic equilibrium with its monomer.^61^^,^^62^ The dimerization is important for a protein's IgE crosslinking capacity.^25^ BLG aggregates upon pasteurization, whereas cooking destroys its antigenicity.^41^ BLG oligomers occurring after dry heating are resistant to reduction.^57^ At high pressure the BLG dimer starts dissociating, and changes in charge and conformation are observed,^63^ either a) a complete protein unfolding, from native dimers to Gaussian chains, or b) a partial unfolding with oligomerization in tetramers mediated by disulfide bridges.^64^ Heating, especially at higher temperatures, is followed by Maillard reaction. While in the “alarmin theory” typically advanced glycated end products (AGEs) support allergenicity,^65^ this is not the case for BLG: heat-mediated aggregation of BLG with milk lactose reduces the antigenicity of BLG as well as the degranulation of mast cells sensitized with specific BLG-IgE.^66^ Glycated BLG is better internalized via galectin-3 (Gal-3) and scavenger receptors (CD36 and SR-A1) into antigen-presenting cells (APCs) than heated BLG.^67^ Other authors showed that aggregation but not glycation is more important for cellular uptake into THP-1 cells.^68^ In its native, conformationally intact form, however, BLG binding to lipocalin-interacting-membrane-receptor (LIMR) has been described,^69^ a necessary requirement for targeted micronutrition to counterbalance allergies.^70^

BLG belongs to the retinoic acid binding family, a sub-family of the lipocalin family, and is characterized by an intramolecular pocket to bind and transport small hydrophobic ligands such as fatty acids, retinoic acid,^71^ vitamin D, cholesterol and aromatic compounds and iron-flavonoid complexes.^70^ BLG is able to bind up to three different ligands simultaneously,^72^ including plant flavonoids and iron-flavonoid complexes, especially in the dimeric state of BLG.^73^ BLG binds to specific receptors on immune cells and by transporting its cargo partakes in establishing immune resilience.^70^^,^^71^ Therefore, BLG in combination with its ligands, so-called holo-BLG, is not an allergen, but a tolerogen.^70^ Its beneficial effect in pollen and house dust mite allergies in preclinical studies^74^^,^^75^ has recently led to the development of a holo-BLG lozenge as a food for specific medical purposes (FSMP). In controlled clinical trials the BLG lozenge imitated the protective farm effect of raw cow's milk and reduced the symptom burden in house dust mite^76^ and pollen allergic patients.^77^

### Immunoglobulins

In bovine milk maternal IgG and IgA are part of the whey fraction. The immunoglobulins are transferred by milk to the calf for passive immune protection against infections, taking advantage of the mother cow's immune repertoire. When human IgE binds to these immunoglobulins they are termed *Bos d 7*. As immunoglobulins are composed of heavy and light chains which are connected by disulfide bonds, they are susceptible to reduction. Moreover, heating above 65 °C destroys the structure of immunoglobulins. This impairs their specific antigen-binding capacity, as well as binding to the receptors via their constant domains. Hence, heated immunoglobulins can only serve as nutrients, but can no longer fulfill their neutralizing function in passive immune defense.

### Serum albumins

Bovine serum albumin (BSA) (pdb 4F5S) is also a whey protein and is termed *Bos d 6*, when recognized by IgE of allergic patients (Table 2). It is a minor allergen. Serum albumins are known to function as a carrier for many bioactive ligands in a 1:1 stoichiometry, eg, lipids, but also binding heme and thereby contributing to reducing heme-cytotoxicity, and, by binding flavonoids like quercetin, enhancing its stability.^78^ A monomeric and dimeric variant are depicted in Fig. 2. Enhanced amounts of albumins are secreted into milk in mastitis of dairy cows. They are one of many biomarkers of inflammation, being important (unfavorable) parameters in milk production.^79^ Serum albumins denature at 72 °C. They are the major responsible of cross-reactivity among milk and beef.^80^

### Lactoferrin

Lactoferrin is dominant in human milk, but in bovine milk present only in minute amounts. Proteins of both species are homologous. Bovine lactoferrin is a 76 kDa glycoprotein, consisting of 2 similar halves (pdb 4OQO). Lactoferrin has a role in iron homeostasis and immune defense: upon intracellular infection, it releases iron into the phagocytic vacuoles for generating reactive oxygen species as defense strategy. Its bioactive peptide of 25 amino acids is lactoferricin, which has an antimicrobial function. Lactoferrin is generally not defined as an allergen,^81^ or only defined as minor allergen even though murine and human studies propose its potential allergenicity (Table 2).

### Proteins of the casein fraction of milk

After acidic coagulation of milk, the casein mass precipitates from the aqueous phase and can be used eg, for cheese production.

### Caseins

Milk caseins form heteromultimers as micelles consisting of αs1-, αs2-, β- and κ-casein. Caseins are precipitated during milk fermentation (Fig. 2). They are very heat-resistant, but digestion-sensitive. When IgE binds to the heteromultimer, it is termed Bos d *8*, whereas the single casein species are named Bos d 9*–12* (Table 2; Fig. 2). Caseins serve as nutrient and casein peptides have many bioactive functions^48^: peptides from α-, β- and κ -caseins are opioid agonists (decrease intestinal mobility; increase uptake of amino acids and electrolytes), ACE-inhibitory (anti-hypertensive). Caseins very efficiently bind minerals such as Ca, P, and Zn (preventing the calcification of the mammary gland, important for bone formation^82^) and have immunomodulatory as well as anti-microbial features; αs1-and αs2-caseins are microbicidic; κ-casein is antithrombotic, and like β-casein also serves as a probiotic (Bifidobacterium growth-activity promoter).

Due to their colloidal properties, they are also added to a large number of food products such as infant food and protein shakes, as well as cosmetics. Caseins are also exploited as carrier for different drugs and pharmaceutical compounds.^83^, ^84^, ^85^

## Pathophysiology

### Mechanisms of IgE-mediated reactions to cow's milk

In cow's milk sensitized patients, IgE fixed to effector cells via the high affinity IgE receptor FcεRI is causative for the appearance of symptoms. Upon secondary encounter of milk proteins, crosslinking of IgE antibodies elicits degranulation of mast cells, basophils or eosinophils. As milk processing renders aggregation of homo- and heteromeric protein complexes, not only their intestinal uptake pathway is directed towards the Peyer's patches,^41^ also their IgE crosslinking capacity can be affected. Typically, symptoms occur immediately, or within minutes to 1–2 h latest. Depending on the level of IgE antibodies, as a measure for the grade of sensitization, different levels of immediate-type symptoms may occur locally, like an oral allergy syndrome with lip and mouth swelling, over pharyngeal swellings and facial angioedema. Also contact urticaria is typically associated only with IgE-mediated CMA.^86^ Upon ingestion of the milk, the smooth muscles in esophagus and stomach can quickly react and cause vomiting, or in the intestine lead to diarrhea (immediate gastrointestinal hypersensitivity). Absorption of milk proteins via the mucosa can occur at any of these time points and potentially lead to systemic reactions, like skin exacerbations (acute urticaria, erythema, angioedema), asthma attacks, and also anaphylactic shock in severely milk allergic patients. Most patients tolerate cooked milk as here the whey proteins, responsible for the majority of allergic reactions, are destroyed, but several reports proposed that the culprit could be also caseins.^87^, ^88^, ^89^, ^90^, ^91^, ^92^

Often, several symptoms occur in combination. The distribution of absorbed milk proteins in the blood stream is enhanced by exercise.

### Mechanisms of mixed IgE-mediated and non-IgE mediated reactions

In the mixed and more delayed type of milk allergic reactions, IgE is a key compound in the pathophysiology, but the inflammation is primarily caused by IgE crosstalk with inflammatory cells. Preformed IgE is not only bound on the IgE effector cells expressing FcεRI, but especially on professional antigen-presenting cells (APCs) like dendritic cells (DCs), where IgE mediates milk allergen uptake, processing and antigen presentation. Thereby, milk peptides with human leucocyte antigen (HLA) class II molecules are expressed on the APCs and recognized by T-cell receptors of CD4^+^ T-helper cells, which release Th2 cytokines such as IL-4 and IL-13, which further drive IgE formation; in addition, released IL-5 recruits eosinophilic granulocytes. Via cross-presentation onto HLA I molecules the APCs can also co-activate CD8^+^ cytotoxic T-cells further promoting the inflammation. Typically, this process of uptake to presentation and T-cell activation takes at least 12–48 h, rendering delayed tissue inflammation.

In the skin, milk in sensitized children may exacerbate **atopic dermatitis**, a mixed inflammatory reaction with CD4^+^ and CD8^+^ T-cell, macrophage, DC and eosinophilic infiltrates, rendering the typical eczematous phenotype. Both skin barrier and microbiota composition are impaired in atopic dermatitis (AD).^93^ There is a strong participation of the innate immune system, as epithelial damages cause the release of IL-31 and IL-33, triggers of severe itch, as well as activation of ILC2 cells, again releasing Th2 cytokines and supporting the IgE pipeline, in terms of specific and total IgE levels.

Repeated allergen exposure leads to sequential inflammation followed by healing processes. The release of autoantigens in inflammation initiates auto-inflammatory processes and, thereby, chronification of the inflammation. Typically, the recruited eosinophils are initiating reconstitution of the skin.^94^ Wound healing mechanisms contribute to the typical thickening of the skin and lichenification in atopic dermatitis.

Specific T-cell infiltrates in the esophagus and stomach of a milk-allergic patient may be triggered by ingested milk antigen and cause the recruitment of eosinophils via IL-5. This process results in **eosinophilic gastrointestinal disorders (EGID)** – ie, **eosinophilic esophagitis**, **eosinophilic gastritis, and eosinophilic enteritis**. In fact, cow's milk has been reported to be the single most important food allergen underlying esophageal inflammation.^95^ In EGID's, it is not clear whether IgE is the first trigger, and whether the accumulating eosinophils in the inflamed tissue may be regarded as an insufficient effort of remodeling.^96^

### Mechanisms of non-IgE mediated reactions to cow's milk

#### Food protein-induced allergic proctocolitis (FPIAP)

Food protein-induced allergic proctocolitis (FPIAP) is an inflammatory disease that mainly affects the distal portions of the colon and is characterized by rectal bleeding.^97^ One study has reported cumulative evidence of up to 17% of newborns;^98^ however its exact prevalence has not been established. It is IgE-independent, but connected with an atopic phenotype and eosinophilia,^99^ but there can be overlaps with IgE-mediated milk allergy and therefore, IgE testing is recommended in infants with AD prior to milk reintroduction.^100^ A recent study demonstrated that exclusive formula feeding is a risk for FPIAP, whereas breast feeding alone or alongside with formula supplementation from birth is protective,^98^ suggesting that passively transferred factors from the mother are pivotal in establishing tolerance. On the other hand, the causative agents for FPIAP are often maternal dietary proteins transferred via breast milk to the baby, and so also in exclusively breastfed infants milk allergy has been observed. This is in part due to the fact that also intact milk proteins can be secreted into the breast milk, for instance, when maternal digestion is hampered by intake of proton pump inhibitors. BLG can serve as a marker for maternal dietary proteins in breast milk as it is not naturally present in human milk (only in the milk of cows, goat, sheep).^100^ FPIAP children have a doubled risk for developing IgE-mediated food allergies later on in life.^100^^,^^101^ In a recent study, cow's milk was by far the most frequent specific allergen (94%), followed by egg, beef, wheat, and nuts, and roughly 43% of diagnosed children were polysensitized to food.^99^ Biopsies are characterized by lymphoid follicles, and cellular infiltrates of lymphocytes as well as eosinophils. The allergic inflammation is associated with enhanced gut permeability supported by dysregulated inflammatory cytokines, elevated pro-inflammatory TNF-α simultaneously to lowered expression of the immunoregulatory TGF-β.^102^ Allergen elimination, also in the maternal diet, is effectively leading to remission.^99^

### Food protein-induced enterocolitis syndrome (FPIES)

The pathophysiology of food protein-induced enterocolitis syndrome (FPIES) involves innate and adaptive immune mechanisms, without IgE involvement.^103^ In up to 50% of cases, cow's milk is the specific cause, followed by oatmeal, rice, soy, wheat and egg. It is believed that the (non-IgE dependent) allergic reaction elevates the intestinal permeability, or vice versa, that enhanced permeability due to genetic factors (atopy) or dysbiosis may be the primary causes. Enhanced permeability leads to an influx of more antigen, which in the context with danger signals leads to innate and adaptive immune activation.^24^ The symptoms, both acute (emesis, diarrhea, lethargy, pallor) and chronic (intermittent, progressive vomiting and diarrhea), are triggered in an antigen-specific manner which can be exploited in diagnosis using food challenges.^104^ In the absence of the causative food the symptoms resolve. Thus, fast and correct diagnosis is important for optimal management strategies to allow thriving of the infant.

Typical laboratory findings in the peripheral blood are leukocytosis with neutrophilia, and thrombocytosis. In cytokine analyses, TNF-α, IL-2, IL-5 and IL-8, and tryptase are elevated, explaining the higher permeability, with T-cell, eosinophil, neutrophil and mast cell infiltrates being present in the intestines of FPIES patients.^105^ Recently, it was discovered that CD4+IL17+ T-cells play a role in the pronounced neutrophilic inflammation via IL-17 release.^106^ Although smaller studies showed antibody formation -especially of the IgG and IgA isotype-upon diagnostic milk challenge, larger studies did not find any correlation.^107^ Only in the 30% of (atypical) patients, elevated Th2 cytokines and specific IgE against the offensive food can be found, but this seems to be predictive for a more persistent, chronic disease.^108^ Typically, in FPIES IgE against food proteins, including milk, is not detected.^109^

### Food protein-induced enteropathy (FPE)

Food protein-induced enteropathy (FPE) targets the small intestine and leads to diarrhea, malabsorption, and protein-loss, and therefore impairs growth. This non-IgE mediated condition may overlap with IgE-mediated milk allergy to food proteins (egg white, cow's milk, wheat and peanut). It can hardly be influenced by maternal elimination diets.^100^

The intestinal inflammation affects the architecture of the crypts and presents with villous blunting, elevated IELs (intraepithelial lymphocytes) and eosinophils.

### Food-protein induced dysmotility disorders and obstipation

In **gastrointestinal esophegeal reflux disorders (GORD)** primarily cow's milk, and to a lesser extent soy, egg and wheat proteins (encountered directly or via breast milk), cause functional obstipation. It is suspected that in the IgE-independent allergic reaction mast cells (besides eosinophils and lymphocytes) interact with gastrointestinal sensitive nerves, rendering motility dysfunction.^100^^,^^110^

### Heiner syndrome

This clinical phenotype is associated with CMA, eosinophilia, high titers of precipitating anti-cowás milk IgG antibodies and high levels of total IgM, IgE, and IgA.^111^ It is characterized by enhanced susceptibility to infections in ear (recurrent otitis media) and respiratory tract (pneumonia and pulmonary hemosiderosis), as well as gastrointestinal symptoms (vomiting, diarrhea, colic, and hematochezia), which resolve after a milk-free diet. Despite iron-deficiency anemia in the blood,^112^^,^^113^ accumulation of iron can occur in tissues and cells, eg, in macrophages.^111^^,^^112^ In rare cases, also hematemesis can occur.^114^ The pathophysiology so far is descriptive, and the mechanism is not revealed yet.

### Dermatitis herpetiformis Duhring (DHD)

Dermatitis herpetiformis Duhring (DHD) is best known from celiac disease, where 30% of patients suffer from herpes-like vesicular eruptions of the skin. In immunofluorescence, immune complex deposits can be detected in the skin, consisting of complement-activating immunoglobulins, IgA and IgG, the specific antigen (gluten) and activated complement. Similarly, DHD can be found in some patients with milk allergy.^115^ It can be hypothesized that enhanced uptake of milk proteins, due to the enhanced intestinal permeability associated with atopy (leaky gut), may lead to IgG or IgA production and immune complex formation and deposition in tissues, depending on the solubility of these complexes. In addition, in Celiac disease the intestinal inflammation may cause secondary lactose intolerance.

### Contact dermatitis

Contact dermatitis to milk is rare and caused by the specific T-cell infiltrates at the dermal exposure site. Their survival may be fostered by macrophage-derived IL-27, which leads to IL-15 production from keratinocytes, an essential survival factor for T-cells.^116^ Contact dermatitis may occur upon skin exposure to milk proteins such as in occupational settings, often when the skin barrier is also impaired due to physical, mechanical or chemical stress.^117^

## Conclusion

Human breastmilk is the optimal nutrition for human babies. It contains immunomodulatory compounds that support tolerance formation to maternal food proteins taken up via breast milk, and to complementary food of the baby. Infant formula from bovine milk is the second-best option, if mother's own milk is not available and is normally introduced after the fourth month, as is complimentary food. Today, commercially available cow's milk is a highly processed product. Harsh manufacturing processes (heating, filtration, pressure, drying, or freezing) negatively affect several beneficial compounds present in fresh, unprocessed cow's milk, which are known to protect from allergies and infections. The protective factors of unprocessed cow's milk are also related to the structurally intact whey protein fraction and the presence of lipophilic ligands. Further, immunoglobulins and cytokines support the tolerability of unprocessed cow's milk. The characteristics of the major single milk proteins are well described today, and milk processing affects them in different ways, causing changes that are immunologically highly relevant (Fig. 1). Processing of milk leads to denaturation of the proteins, exposure of new antigenic epitopes, and destruction of the contained immunomodulatory factors and cytokines. In addition, defatting processes remove protective lipophilic ligands (retinoic acid, flavonoids), and complexes thereof. The baby may encounter milk proteins directly, or derived from the maternal diet through the breast milk. Aggregated milk proteins are directed to the Peyer's patches, the active immune induction sites in the terminal ileum, leading to specific milk-adverse reactions. The manifold antigenic and immunogenic changes of processed caseins and whey proteins, such as ALA, BLG, BSA, lactoferrin, immunoglobulins and others, are reflected by a spectrum of adverse reactions to milk, including A) immediate type IgE-mediated, B) mixed, and C) delayed type immunotoxic reactions independent of IgE. We need to better understand the molecular events during dairy processing to completely capture the resulting subsequent immune mechanisms. Next generation dairy techniques should aim at avoiding that sensitizing molecules develop in the process.

## Abbreviations

AAMP, allergen-associated molecular patterns; AGEs, glycated end products; ALA, alpha-lactalbumin, α-lactalbumin; ALP, alkaline phosphatase; APCs, antigen-presenting cells; BAMLET, Bovine Alpha-Lactalbumin Made Lethal to Tumor cell; BCF, bovine colostrum fortifier; BLG, beta-lactoglobulin, β-lactoglobulin; BMF, bovine-derived milk fortifiers; BSA, bovine serum albumin; CMP, cow's milk protein; CMA, cow's milk allergen; CMPA, cow's milk protein allergy; DCs, dendritic cells; DHD, Dermatitis Herpetiformis Duhring; DRACMA, Diagnosis and RAtionale against Cow's Milk Allergy; EAACI European Academy of Allergy and Clinical Immunology; ESL, extended lifetime; FPIAP, Food protein-induced allergic proctocolitis; FPIES, Food protein-induced enterocolitis syndrome; Gal-3, galectin-3; GORD, gastrointestinal oesophegeal reflux disorders; HAMLET, Human Alpha-Lactalbumin Made Lethal to Tumor cell; HCl, hydrochloric acid; HLA, human leucocyte antigen; HMF, human milk-derived fortifiers; IELs, intraepithelial lymphocytes; IL-, interleukin; ILC2, innate lymphoid cells 2; Lf, Lactoferrin; LIMR, lipocalin-interacting-membrane-receptor; LPS, lipopolysaccharide; LTLT, low temperature/long time; MD-2, myeloid differentiation-2 molecule; NFkB, Nuclear factor kappa B; NMR, nuclear magnetic resonance; PAR, protease-activated receptors; PRR, pattern-recognition receptors; SR-A1, scavenger receptor class A1; STHT, short time/high temperature; TGF-β, transforming growth factor-beta; TLR4, Toll-like receptor-4; TNF-α, tumor necrosis factor-alpha.

## Funding

SAJ, FRW and EJJ were supported by the Danube Allergy Research Cluster (10.13039/501100002958DARC), project #08 by the Karl Landsteiner University, Krems, Austria. EJJ, 10.13039/100017393CLP and 10.13039/100011090IPS were supported by the Swiss 10.13039/501100011567Messerli Foundation. 10.13039/100017393CLP was supported Austrian Science fund project W1248-B30 to EJJ within MCCA (Molecular, Cellular and Clinical Allergology) doctoral program of the Medical University Vienna; TB is funded by the Software AG Stiftung, Darmstadt, Germany. There was no external source of funding obtained for this study.

The World Allergy Organization is the funder of the WAO DRACMA guidelines update project.

## Availability of data and material

Not applicable.

## Authors' contributions

SAJ drafted the manuscript and searched the literature. EJJ supervised and guided the research. EJJ, IPS, GJ, AGF and FRW critically revised the manuscript and contributed to the figure design. GJ designed and art worked Fig. 1. TB, ANW, SP, KT, AHA, CLP and CV contributed to the writing and critically reviewed the paper.

**Fig. 1:**
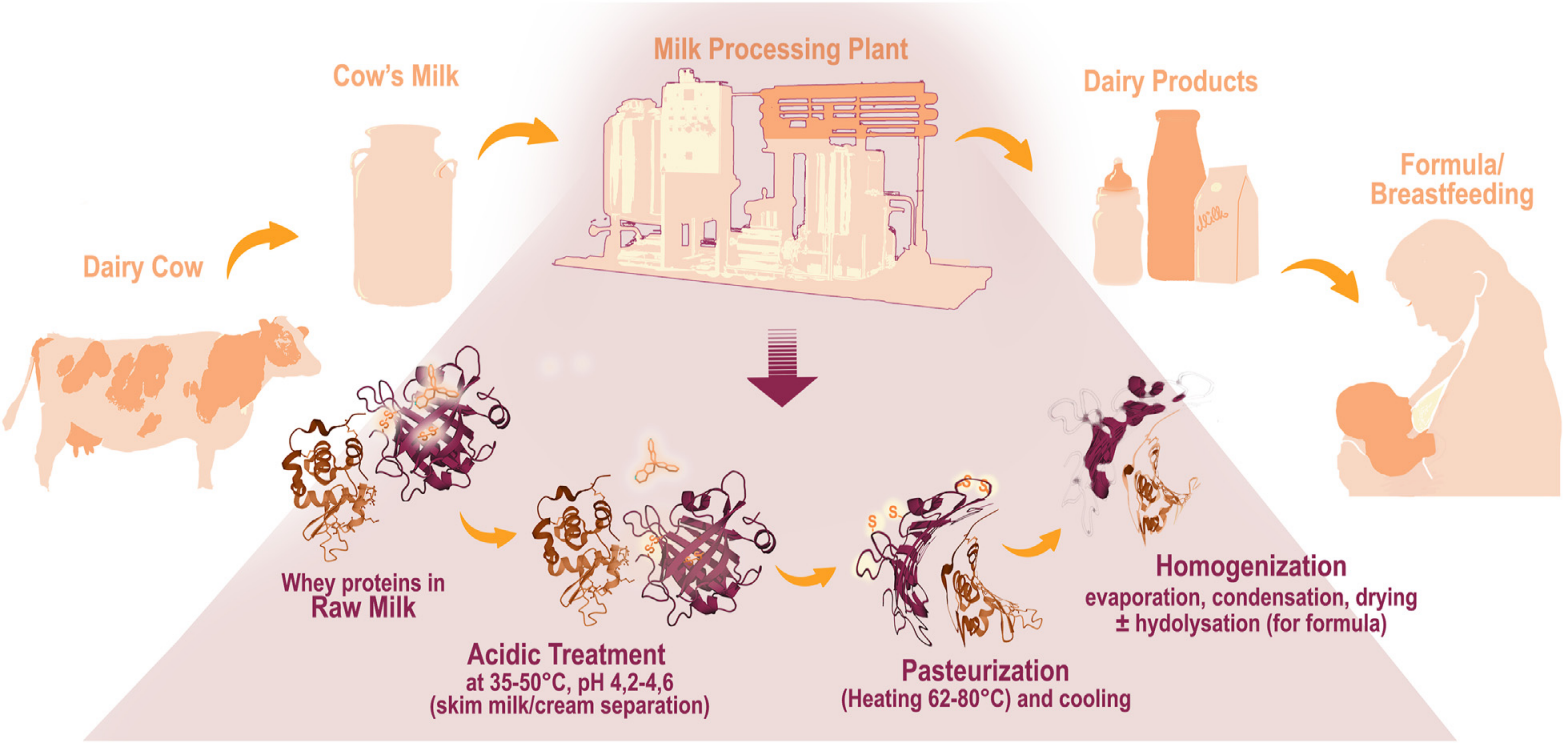
**Milk processing changes the 3D structure of whey proteins**. Top: Cow's milk is an essential food and needs to be distributed to the people around the world. Therefore, milk processing aims at a) avoiding zoonotic infections of the consumer, and b) making milk products transportable and expanding their shelf life time. Milk processing plants are equipped to fulfill these needs and deliver safe products suited for all tastes and demands, from milk, to infant formula, and many more. Bottom: The major whey proteins are beta-lactoglobulin (50%–65% of all whey proteins) (colored dark red) and alpha-lactalbumin (10–12%) (colored orange). Derived from the dairy cow, these proteins are conformationally intact, and emulsified together with lipophilic compounds and vitamins in raw farm milk. Several processing steps in a dairy plant significantly impair the 3D-structure of whey proteins, their homo- and heteromeric aggregation state, the composition of all hydrophilic and lipophilic milk constituents, and thereby change the immunogenicity and allergenicity of milk

## Ethics approval and consent to participate

Not applicable.

## Consent for publication

All authors agreed to publication of the work.

## Declaration of competing interest

EJJ and FRW declare inventorship on EP2894478 (“Lipocalins for AIT”), owned by Biomedical International R + D, Vienna, Austria, of whom EJJ is shareholder. TB performs limited paid consulting on dairy farms and presentations at workshops for raw milk producers; CV reports grants from Reckitt, personal fees from Reckitt, personal fees from 10.13039/100007773Danone, personal fees from Abbott, personal fees from Nestle Nutrition Institute, personal fees from Sifter, outside the submitted work. The other authors declare that they have no competing interests.

## Members of the WAO DRACMA guideline group:

Ignacio J. Ansotegui, MD, PhD (Department of Allergy & Immunology, Hospital Quironsalud Bizkaia, Erandio, Bilbao, Spain); Stefania Arasi, MD, PhD (Translational Research in Pediatric Specialities Area, Division of Allergy, Bambino Gesù Children’s Hospital, IRCCS, Rome, Italy); Amal H. Assa’ad, MD (Division of Allergy and Immunology, Cincinnati Children’s Hospital Medical Center, Cincinnati, OH, USA); Sami L. Bahna, MD, DrPH (Allergy/Immunology Section, Louisiana State University Health Sciences Center, Shreveport, LA, USA); Roberto Berni Canani, MD, PhD (Department of Translational Medical Science, University of Naples Federico II, Naples, Italy); Antonio Bognanni, MD (Department of Health Research Methods, Evidence and Impact - HEI, McMaster University, Hamilton, ON, Canada); Martin Bozzola, MD (Department of Pediatrics, Pediatric Allergy/Immunology Section, British Hospital, Buenos Aires, Argentina); Jan Brozek, MD, PhD (Department of Medicine, Division of _ Clinical Immunology and Allergy, Department of Clinical Epidemiology & Biostatistics, McMaster University Health Sciences Centre, Hamilton, ON, Canada); Derek K. Chu, MD, PhD (Department of Medicine, Division of Clinical Immunology and Allergy; Department of Clinical Epidemiology & Biostatistics, McMaster University Health Sciences Centre, Hamilton, ON, Canada); Lamia Dahdah, MD (Translational Research in Pediatric Specialities Area, Division of Allergy, Bambino Gesù Children’s Hospital, IRCCS, Rome, Italy); Christophe Dupont, MD, PhD (Paris Descartes University, Pediatric Gastroenterology, Necker Hospital, Paris, Clinique Marcel Sembat, Boulogne-Billancourt, France); Motohiro Ebisawa, MD, PhD (Clinical Research Center for Allergy and Rheumatology, National Hospital Organization Sagamihara National Hospital, Kanagawa, Japan); Alessandro Fiocchi, MD (Translational Research in Pediatric Specialities Area, Division of Allergy, Bambino Gesù Children’s Hospital, IRCCS, Rome, Italy); Ramon Targino Firmino MD (Faculty of Medical Sciences of Campina Grande, UNIFACISA University Centre, Campina Grande, Paraiba, Brazil); Elena Galli, MD, PhD (Pediatric Allergy Unit, Research Center, San PietroFatebenefratelli Hospital, Rome, Italy); Rose Kamenwa, MD (Department of Pediatrics and Child Health, Aga Khan University Hospital, Nairobi, Kenya); Gideon Lack, MBBCh (Department of Women and Children’s Health/Peter Gorer Department of Immunobiology, School of Life Course Sciences, Faculty of Life Sciences & Medicine, King’s College London, UK; Evelina London Children’s Hospital, Guy’s and St Thomas’ Hospital NHS Foundation Trust, London, UK), Haiqi Li, MD (Pediatric Division Department of Primary Child Care, Children’s Hospital, Chongqing Medical University, Chongqing, China); Alberto Martelli, MD (Italian Society of Pediatric Allergy and Immunology, Milano, Italy); Anna H. Nowak-Wegrzyn, MD, PhD (Department of Pediatrics, New York University Langone Health, New York, NY, USA; Department of Pediatrics, Gastroenterology and Nutrition, Collegium Medicum, University of Warmia and Mazury, Olsztyn, Poland); Nikolaos G. Papadopoulos, MD, PhD (Allergy Unit, 2nd Pediatric Clinic, University of Athens, Athens, Greece; Division of Infection, Immunity & Respiratory Medicine, University of Manchester, UK); Ruby Pawankar, MD, PhD (Department of Pediatrics, Nippon Medical School, Bunkyo-Ku, Tokyo, Japan); Maria Said, RN (Allergy & Anaphylaxis Australia (A&AA), Castle Hills, New South Wales, Australia); Mario Sánchez-Borges MD (Department of Allergy and Clinical Immunology, Centro Médico-Docente La Trinidad Caracas, Venezuela); Holger J. Schünemann, MD, MSc, PhD (Department of Health Research Methods, Evidence and Impact (HEI), McMaster University, Hamilton, ON, Canada, and Cochrane Canada and McMaster GRADE Centre, Hamilton, ON, Canada); Raanan Shamir, MD, PhD (Institute of Gastroenterology, Nutrition and Liver Disease, Schneider Children’s Medical Center, Petach-Tikva, Israel; Sackler Faculty of Medicine, Tel-Aviv University, Tel-Aviv, Israel); Jonathan M. Spergel, MD, PhD (Division of Allergy and Immunology, Department of Pediatrics, The Children’s Hospital of Philadelphia, Perelman School of Medicine at University of Pennsylvania, Philadelphia, PA, USA), Hania Szajewska, MD (The Medical University of Warsaw - Department of Paediatrics, Warsaw, Poland); Luigi Terracciano, MD (Italian NHS and Italian Society of Social and Preventive Pediatrics, Milano, Italy); Yvan Vandenplas, MD, PhD (Department of Pediatrics, UZ Brussel, Vrije Universiteit Brussel, Brussels, Belgium); Carina Venter, PhD, RD (Section of Allergy & Immunology, University of Colorado Denver School of Medicine, Children’s Hospital Colorado, Aurora, CO, USA); Amena Warner, RN, SN (PG Dip) (Allergy UK, Planwell House, Sidcup, Kent, UK); Susan Waserman, MD, MSc (Division of Clinical Immunology and Allergy, Department of Medicine, McMaster University, Hamilton, ON, Canada); Gary W. K. Wong, MD (Department of Paediatrics, Faculty of Medicine, The Chinese University of Hong Kong, Hong Kong, China).

## WAO DRACMA guideline group – Declarations:

S Arasi, S Bahna, Bognanni, J Brozek, D Chu, L Dahdah, E Galli, R Kamenwa, H Li, A Martelli, R Pawankar, H Schunemann, R Targino, L Terracciano, and A Warner have no conflicts to disclose. Relationships reported related to the submitted work: IJ Anstotegui – Abbott, Amgen, Astra Zeneca, Bayer, Bial, Faes Farma, Hikma, Menarini, Merck, Mundipharma, Roxall, Sanofi, Stallergenes, UCB. A Assa’ad – Aimmune Therapeutics, DBV Technologies, Astella, ABBVIE, Novartis, Sanofi, FARE, NIH and an intellectual property patent licensed to Hoth. R Berni Canani – Ch.Hansen, Danone, DVB, Humana, iHealth, Kraft Heinz, Mead Johnson, Nestlè, Novalac, Nutricia, Sanofi. M Bozzola – Danone C Dupont – Nestle Health Science, Nestle France, Nutricia, Novalac, Sodilac, Abbott, Danone, and stock ownership at DBV Technologies. M Ebisawa – DBV Technologies, Mylan, ARS Pharmaceuticals, Novartis. A Fiocchi – Abbott, Danone. G Lack – FARE, National Peanut Board (NPB), The Davis Foundation, Action Medical Research, UK Food Standards Agency, Medical Research Council, DBV Technologies, Mission Mighty Me, Novartis, Sanofi-Genyzme, Regeneron, ALK-Abello, Lurie Children’s Hospital. A Nowak-Wegrzyn – Nestle, Nutricia, Novartis, Gerber, Aimmune. N Papadopoulos – Novartis, Nutricia, HAL Allergy, Menarini/ Faes Farma, Sanofi, Mylan/Meda, Biomay, AstraZeneca, GSK, MSD, ASIT Biotech, Boehringer Ingelheim, Gerolymatos International SA, Capricare. M Said – Nestle, Nutricia, Abbott, Bayer for Anaphylaxis Australia. J Spergel – DBV Technologies, Regeneron, Sanofi, and Aimmune. H Szajewska – Ausnutria, Cargill, Danone, Else Nutrition, Hipp, Nestle, and Nestle Nutrition Institute. Y Vandenplas – Abbott Nutrition, Biogaia, Biocodex, By Heart, CHR Hansen, Danone, ELSE Nutrition, Friesland Campina, Hero, Hypocrata, Nestle Health Science, Nestle Nutrition Institute, Nutricia, Mead Johnson Nutrition, Orafti, Phacobel, Phathom Pharmaceuticals, Sari Husada, United Pharmaceuticals (Novalac), Wyeth, Yakult. C Venter – Reckitt Benckiser, Nestle Nutrition Institute, Danone, Abbott Nutrition, Else Nutrition, and Before Brands, DBV Technologies. S Waserman – Novartis-basic science work on peanut allergy, Aimmune-peanut OIT trial, Medical Advisor to Food Allergy Canada, and Pfizer, Bausch, Kaleoconsultant for epinephrine autoinjectors. GWK Wong – Nestle, Danone.

## Figures and Tables

**figs1:**
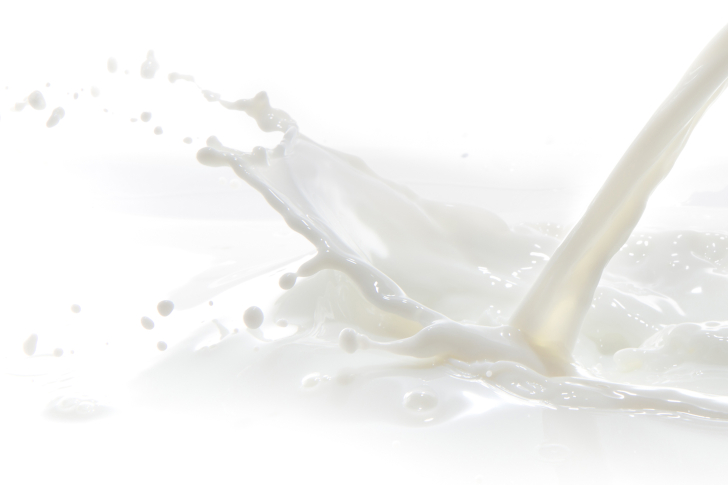


**figs2:**
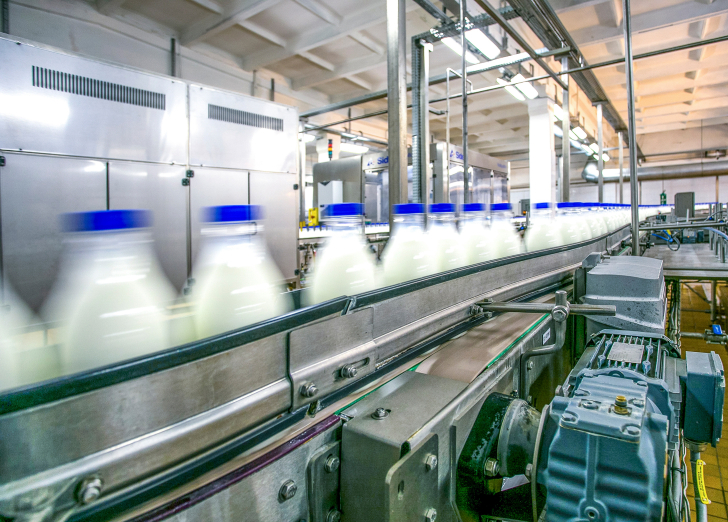


**figs3:**